# Capturing seizures in clinical trials of antiseizure medications for *KCNQ2*‐DEE

**DOI:** 10.1002/epi4.12466

**Published:** 2021-01-29

**Authors:** John J. Millichap, Cynthia L. Harden, Dennis J. Dlugos, Jacqueline A. French, Noam N. Butterfield, Celene Grayson, Ernesto Aycardi, Simon N. Pimstone

**Affiliations:** ^1^ Epilepsy Center and Division of Neurology Ann & Robert H. Lurie Children’s Hospital of Chicago Chicago IL USA; ^2^ Departments of Pediatrics and Neurology Northwestern University Feinberg School of Medicine Chicago IL USA; ^3^ Xenon Pharmaceuticals, Inc. Burnaby British Columbia Canada; ^4^ Children's Hospital of Philadelphia Perelman School of Medicine at the University of Pennsylvania Philadelphia PA USA; ^5^ Department of Neurology NYU Langone School of Medicine New York NY USA; ^6^ Division of General Internal Medicine University of British Columbia Burnaby British Columbia Canada

**Keywords:** epilepsy, KCNQ2, seizure diaries, tonic seizures, video‐EEG

## Abstract

Literature review of patients with *KCNQ2* developmental and epileptic encephalopathy (*KCNQ2*‐DEE) reveals, based on 16 reports including 139 patients, a clinical phenotype that includes age‐ and disease‐specific stereotyped seizures. The typical seizure type of *KCNQ2*‐DEE, focal tonic, starts within 0‐5 days of life and is readily captured by video‐electroencephalography VEEG for clinical and genetic diagnosis. After initial identification, *KCNQ2*‐DEE seizures are clinically apparent and can be clearly identified without the use of EEG or VEEG. Therefore, we propose that the 2019 recommendations from the International League against Epilepsy (ILAE), the Pediatric Epilepsy Research Consortium (PERC), for capturing and recording seizures for clinical trials (*Epilepsia Open*, 4, 2019, 537) are suitable for use in *KCNQ2*‐DEE‒associated antiseizure medicine (ASM) treatment trials. The ILAE/PERC consensus guidance states that a caregiver‐maintained seizure diary, completed by caregivers who are trained to recognize seizures using within‐patient historical recordings, accurately captures seizures prospectively in a clinical trial. An alternative approach historically endorsed by the Food and Drug Administration (FDA) compares seizure counts captured on VEEG before and after treatment. A major advantage of the ILAE/PERC strategy is that it expands the numbers of eligible patients who meet inclusion criteria of clinical trials while maintaining accurate seizure counts (*Epilepsia Open*, 4, 2019, 537). Three recent phase 3 pivotal pediatric trials investigating ASMs to treat syndromic seizures in patients as young as 2 years of age (*N Engl J Med*, 17, 2017, 699; *Lancet*, 21, 2020, 2243; *Lancet*, 17, 2018, 1085); and ongoing phase 2 open‐label pediatric clinical trial that includes pediatric epileptic syndromes as young as 1 month of age (*Am J Med Genet A*, 176, 2018, 773), have already used caregiver‐maintained seizure diaries successfully. For determining the outcome of a *KCNQ2*‐DEE ASM treatment trial, the use of a seizure diary to count seizures by trained observers is feasible because the seizures of *KCNQ2*‐DEE are clinically apparent. This strategy is supported by successful precedent in clinical trials in similar age groups and has the endorsement of the international pediatric epilepsy community.

**Key points**

- Loss‐of‐function pathogenic variants in the *KCNQ2* gene cause a developmental epileptic encephalopathy.
- Seizure counting in clinical trials of antiseizure medications in pediatrics is likely best accomplished without using video‐EEG to count seizures.
- The typical seizures of *KCNQ2*‐DEE are focal tonic and accurately recognizable by caregivers.

## 1 POSITION

The purpose of this paper is to provide justification for using a caregiver‐maintained seizure diary, informed by VEEG training, to capture seizure frequency as the primary outcome in *KCNQ2*‐DEE ASM clinical trials. The approach proposed herein follows from the recommendation published in 2019 by the regulatory task force and the pediatric commission of the ILAE, in collaboration with PERC.^1^ We propose that historical VEEG be used to confirm seizures is feasible in *KCNQ2*‐DEE. These recordings would then be used to train caregivers to identify the seizures, enabling them to maintain a seizure diary throughout the clinical trial. For consistency, all efforts would be made to the limit the number of caregiver recording seizures into the diary during the clinical trial.

## 2 BACKGROUND

### 2.1 VEEG is appropriate for primary assessment of seizures in a subset of neonates who have very frequent seizures of heterogeneous etiologies

VEEG has been considered by the Food and Drug Administration (FDA) to be the preferred method to measure seizure frequency in circumstances where seizure counting by observation is problematic. VEEG has been used as a primary outcome measure in ASM trials in infants and young children, from 1 month to 4 years of age.^2^, ^3^, ^4^, ^5^, ^6^, ^7^ The rationale is that some types of seizures in young children may be difficult to distinguish from other nonepileptic movements, making seizure classification and counting difficult. Counting by clinical observation alone could lead to observers counting nonseizure movements as seizures and/or undercounting real seizures. To address these difficulties, the Agency has recommended the use of video‐EEG with correlation of seizures with EEG electrical activity for study of infants and neonates. For treatment trials that include infants,^2^, ^3^, ^4^, ^5^, ^6^, ^7^ video‐EEG has often been performed for 48 to 72 hours before treatment with review by several expert reviewers to achieve consensus on the seizure types and frequency. Following treatment with an ASM, another video‐EEG over the same time frame is performed and the seizure counts compared to pretreatment observations to determine the primary endpoint of seizure reduction. While treatment trials are less common in the neonatal period, evaluating seizures in neonates presents similar challenges. Evaluating a treatment by counting seizures captured on video‐EEG both before and after treatment is clearly necessary for evaluating seizures of neonates wherein the etiology is often hypoxic‐ischemic encephalopathy or structural / metabolic, resulting in seizure types that are varied, may be subtle and difficult to identify, and therefore difficult to count without the use of video‐EEG.^8^, ^9^ Further, seizures in children outside the neonatal period are usually clinically apparent, mitigating the need for video‐EEG to inform an accurate seizure count.

### 2.2 Seizures in KCNQ2‐DEE

A literature search of PubMed was undertaken using the key words KCNQ2 encephalopathy, KCNQ2 developmental encephalopathy, KCNQ2 seizures, KCNQ2 developmental and epileptic encephalopathy, KCNQ2 epileptic encephalopathy, KCNQ2‐EEG and KCNQ2‐DEE. Reports from 2012 and on were included as the disease was first described and characterized in 2012 although unrecognized cases were likely described prior to this. Abstracts were not included. Reports with seizure descriptions were included. Literature review of 16 case studies of patients diagnosed with the genetic syndrome of *KCNQ2*‐DEE reveals that the associated seizures are characterized primarily by motor seizure types, focal tonic seizures being the most common, which are present at onset and persist beyond the neonatal period (see Table 1).^8^, ^9^, ^10^, ^11^, ^12^, ^13^, ^14^, ^15^, ^16^, ^17^, ^18^, ^19^, ^20^, ^21^, ^22^, ^23^ Tonic‐clonic and myoclonic seizures are also described. Many seizure descriptions include apnea as a prominent associated feature of the tonic seizure. Other seizure descriptions include generalized tonic‐clonic, generalized tonic, focal motor, myoclonic, and epileptic spasms, which are all clinically apparent motor seizure phenomena. Overall, out of 137 *KCNQ2*‐DEE patients reported in the 16 cited studies, 94% (129 patients) had clear motor seizures (excluding patients with myoclonic events only; see explanation to follow), with the majority of patients having focal tonic seizures (81%, n = 111). In fact, focal tonic seizures were the main seizure type in most, if not all, of the patients described in the cited studies (see Table 1).

**TABLE 1:** Literature review—seizure types observed in patients with *KCNQ2*‐DEE

(Author/Year)	Patients (n)	Motor seizures^a^ (Patients, n)	Total focal tonic (Patients, n)
Duan et al (2018)^8^	1	1	1
Hortiguela et al (2017)^9^	13	13	9
Kato et al (2013)^10^	12	11	11
Ko et al (2018)^11^	7	7	7
Lee et al (2019)^12^	7	7	7
Milh et al (2013)^13^	16	14	12
Millichap et al (2016)^14^	23	19	15
Numis et al (2014)^15^	3	3	3
Olson et al (2017)^16^	10	10	6
Pisano et al (2015)^17^	15	15	12
Schubert‐Bast et al (2017)^18^	1	1	1
Serino et al (2013)^19^	1	1	1
Spagnoli et al (2018)^20^	3	2	2
Vilan et al (2017)^21^	9	9	9
Weckhuysen et al (2012)^22^	8	8	8
Weckhuysen et al (2013)^23^	9 with severe DEE	9	8
	Total N = 137^b^	129^b^/137 (94.2%)	111^b^/137 (81.0%)

^a^ Including focal tonic, generalized tonic, clonic, tonic‐clonic, generalized tonic‐clonic seizures.

^b^ One patient (who had focal tonic seizures) was reported in both Millichap 2016 and Olsen 2017. That patient is only counted once; therefore, the totals in each column appear to be one patient less than when patients from all the reports are added together.

A focal tonic seizure is an electro‐clinical seizure type that is readily observed and associated with a clear EEG correlate of contralateral amplitude suppression at the outset. A description of the characteristic seizures and EEG findings in a 6‐week‐old boy with *KCNQ2*‐DEE is as follows, reported by Serino et al^19^: “Seizure semiology was characterized by unilateral eye and head deviation, upper limb hypertonus sometimes followed by asynchronous and asymmetrical clonic jerks, eyelid myoclonias, and polypnoea. Ictal EEG was characterized by focal, low‐voltage, fast activity, followed by recruiting theta rhythms and bilateral, focal, spike‐wave complexes, alternatively localized to one hemisphere and subsequently diffusing to the other.” A description from Vilan et al^21^ of seizures in nine *KCNQ2*‐DEE infants under 1 month of age states, “Seizures were clinically evident in all patients, characterized by asymmetric tonic posturing accompanied by cyanosis, and at times followed by unilateral clonic activity.”

Notably, there were no reports of electrographic subclinical seizures in the *KCNQ2*‐DEE cases reviewed. Multifocal epileptiform features were frequently described as part of the EEG background, as was an intermittent non‐seizure‒associated burst‐suppression pattern often present until 6 months of age. A minority of patients with *KCNQ2*‐DEE also suffer bouts of epileptic spasms within the first year of life.

In summary, the characteristic seizures of *KCNQ2*‐DEE are clear countable motor events that can be readily clinically observed. The majority are focal tonic, along with other motor seizure types including generalized tonic, clonic, and generalized tonic‐clonic seizures. One exception is myoclonic seizures. Single myoclonic jerks may be nonepileptic and are very hard to count. In many trials, myoclonic jerks are not considered a countable seizure and are not included in the efficacy endpoint. Repeated myoclonic movements are counted as clonic seizures. Finally, it is clear that *KCNQ2*‐DEE affected infants do not have highly variable seizure types or subtle subclinical seizures.

## 3 RATIONALE SUPPORTING USE OF A CAREGIVER DIARY FOR PRIMARY ASSESSMENT OF SEIZURE FREQUENCY IN KCNQ2‐DEE

Given that patients will have had EEG or VEEG confirmation of seizures, which is recommended by the ILAE for the evaluation of children with undiagnosed repeated abnormal events,^24^ the use of seizure diaries as the primary method to evaluate seizure frequency in the infantile and early childhood KCNQ2‐DEE population is supported by the following:

- The most frequent and typical seizures of *KCNQ2*‐DEE are easily observable, most clinically relevant, and easily counted.
  - Multiple reports describe *KCNQ2*‐DEE seizures as stereotypical, most often focal tonic in semiology, and observable from birth.^8^, ^9^, ^10^, ^11^, ^12^, ^13^, ^14^, ^15^, ^16^, ^17^, ^18^, ^19^, ^20^, ^21^, ^22^, ^23^
  - Parents have the ability to correctly identify seizures with a high degree of confidence (*KCNQ2* survey), which can be further improved with training.^1^
- Seizure diaries allow frequent, daily recording of seizure burden to measure treatment effects.
  - This is important because there is typically some variability in the timing and frequency of seizures, so daily seizure capture provides the granularity needed to accurately measure treatment effect.
  - The alternative, VEEG, would only allow for 2‐3 days of seizure capture after treatment.
- Caregiver‐reported seizure diaries have been successfully employed in other studies of pediatric epilepsy syndromes such as Dravet and Lennox‐Gastaut (LGS).
  - For example, in a study of patients with Dravet syndrome, “convulsive seizures” specifically tonic‐clonic, tonic, clonic, or atonic were counted toward the primary outcome.^25^ “Non‐convulsive seizures,” defined as myoclonic, countable focal, other focal, or absence seizures, were captured as a secondary outcome.
  - In another study of Dravet syndrome, again “convulsive” seizures were used to assess the primary outcome, which is a clearly observable and dangerous seizure type.^26^
  - In a study of LGS, “drop seizures” comprised of tonic‐clonic, tonic, or atonic seizures that resulted in the patient dropping, informed the primary outcome.^27^ Total seizures (the sum of all tonic‐clonic, tonic, atonic, clonic, myoclonic, countable focal, other focal and absence seizures) were counted as part of the study's secondary outcomes.
- The ability to use a seizure diary rather than VEEG has many advantages, not the least of which is convenience for the participating families.^1^
  - Subjects in the previously preferred infant trial design were typically required to undergo at least two 24‐48 hour VEEGs, most likely as an inpatient, which is burdensome to families. A 24‐ to 48‐hour VEEG study may not capture enough seizures (or any seizures in patients with less frequent than daily seizures) and therefore may not adequately reflect the true seizure burden of the participant. Alternatively, a seizure diary enables enrollment of patients whose seizure frequency is variable, less than daily, or presents in clusters.^1^

## 4 SEIZURE COUNTING IN AN ASM TREATMENT TRIAL IN KCNQ2‐DEE

For research purposes, the seizure types used for evaluating the effect of an intervention must be clearly defined. As listed in Table 1,^8^, ^9^, ^10^, ^11^, ^12^, ^13^, ^14^, ^15^, ^16^, ^17^, ^18^, ^19^, ^20^, ^21^, ^22^, ^23^ the seizures seen in *KCNQ2*‐DEE include the following motor seizures: focal tonic (most common), generalized tonic, clonic, and generalized tonic‐clonic seizures. These seizure types would be counted in the primary analysis for a study evaluating ASM treatment in *KCNQ2*‐DEE. Important exclusions to countable seizures for *KCNQ2*‐DEE would include single myoclonic jerks or staring spells without a motor component consistent with the seizure types included.

Reports of EEG correlation with clinical seizures support both focal and generalized onset of these seizure types. The EEG background may be chaotic, showing multifocal spikes or burst suppression.^21^, ^22^ Therefore, each seizure type counted in a clinical trial of ASM treatment for *KCNQ2*‐DEE could be classified as either focal or generalized in onset, as supported by EEG correlation.^28^

As part of determining eligibility for an ASM clinical trial in *KCNQ2*‐DEE, an adjudication committee consisting of *KCNQ2* variant experts would need to vet each potential trial participant prior to enrollment by evaluating phenotype, genotype, and VEEG findings. The adjudication committee would provide an additional review of the VEEG information to ensure that appropriate patients are enrolled.

## 5 TRAINING CAREGIVERS ON COUNTING SEIZURES FOR KCNQ2‐DEE CLINICAL TRIALS

A systematic training program to teach caregivers how to identify seizures is proposed below.

1. Locate historical VEEG of typical seizures, or repeat VEEG if seizure semiology has changed; review with parents or caregivers to confirm that these are typical seizures.
2. Invite parents or caregivers to review VEEG results at a time when everyone is available for 1 hour and is calm.
3. Choose no more than two persons to be officially trained as seizure‐counters.
4. Review seizures to be counted in the study via VEEG.
5. Discuss seizure features with seizure‐counters, including what not to count:
  - *KCNQ2*‐DEE seizures include the following motor seizures: focal tonic (most common), generalized tonic, clonic, and generalized tonic‐clonic seizures.
  - Important exclusions to study countable seizures are single myoclonic jerks or staring spells without a motor component consistent with the seizure types included.
6. Play video of myoclonus or other nonseizure events to demonstrate what not to count (within the patient or historical).

## 6 CONCLUSIONS

The use of seizure diaries as the primary method to evaluate seizure frequency in the infantile KCNQ2‐DEE population is supported by the following rationale. Multiple reports conclude that KCNQ2‐DEE seizures are stereotyped and observable from birth. It follows then that seizure diaries are a robust method to capture the true seizure burden of a patient rather than relying on a very small window of time with the use of video‐EEG, and there is precedent for this approach in syndromic epilepsies. Moreover, this approach allows a broader range of patients to be eligible to participate in this important trial, such as those with less than daily seizures in whom video‐EEG as part of the study protocol becomes impractical. Inclusion of a broader group of patients is also key given the rarity of the disease.

Accordingly, we propose that the primary outcome measure of an ASM treatment trial in KCNQ2‐DEE that includes children as young as 1 month of age can be identified and documented by parents in a diary. In a clinical trial setting, an adjudication committee consisting of KCNQ2 variant experts will vet each potential subject for trial enrollment by evaluation of phenotype, genotype, and video‐EEG findings. Training on seizure identification using video‐EEG would be provided systematically to all potential caregivers of a given subject. The seizures captured and used for training parents will be recorded in a seizure diary.

In addition to being supported by multiple preceding studies in pediatric syndromic epilepsies in the age group included in our trial, our proposal is in alignment with a recently published consensus document from the Regulatory Task Force and the Pediatric Commission of the International League against Epilepsy (ILAE), in collaboration with the Pediatric Epilepsy Research Consortium (PERC).^1^ This publication clearly outlines the rational for assessing the outcome of an ASM treatment trial in very young children by using daily outpatient seizure diaries with the seizures to be captured identified by prior EEG, video, and video‐EEG. This approach combines the rigor of accurate seizure identification and classification via video‐EEG with a clinically relevant seizure diary count by caregivers and is practical, efficient, and generalizable to clinical practice.^1^ The consensus statement recommends the following procedure for seizure documentation in an interventional study: “*Seizures with clinical signs will be confirmed by video‐EEG (preferred) or video (with clear semiology and supporting clinical and interictal EEG data) for each study participant and reviewed centrally by experts in pediatric epilepsy. If available, prior clinically obtained video‐EEG or video recordings may be used to confirm that the events in question are seizures if the seizure semiology has remained the same. If no clinically obtained recordings are available, then they will be obtained as study procedure before randomization. The clinically observable focal onset seizures documented by video‐EEG or video will be the events of interest for the study's primary endpoint. In the pretreatment baseline period, titration phase, and maintenance period, the above clinically observable seizures will be recorded in seizure diaries by caregivers.”*

## CONFLICTS OF INTEREST

Drs. Grayson, Harden, Butterfield, Pimstone, and Aycardi are employed by Xenon Pharmaceuticals Inc (“Xenon”). They receive salaries and may hold stock or stock options in Xenon. Dr Millichap reports personal fees from American Academy of Neurology, personal fees from Up‐To‐Date, personal fees from BMJ Best Practice, grants and personal fees from UCB Pharma, grants and personal fees from Mallinkrodt, personal fees from Esai, personal fees from Biomarin, personal fees from Ionis, personal fees from Greenwich, personal fees from Sunovion, personal fees from Upsher‐Smith, grants from NIH, grants from Citizens United for Research in Epilepsy, personal fees from Praxis, grants and personal fees from Trumacro, outside the submitted work; Pursuant to a Consulting Agreement between Ann and Robert H. Lurie Children's Hospital of Chicago and Xenon, it is anticipated that Dr Millichap will serve as the Global Coordinating Investigator for Xenon's planned Phase 3 study of XEN496. The Ann and Robert H. Lurie Children's Hospital of Chicago receives financial compensation from Xenon in exchange for Dr Millichap's services. In addition, Dr Millichap, in a personal capacity, is a consultant for Xenon and also serves as a member the Xenon Steering Committee for XEN496. Dr Millichap receives financial compensation from Xenon in exchange for his services. Dr French receives NYU salary support from the Epilepsy Foundation and for consulting work and/or attending Scientific Advisory Boards on behalf of the Epilepsy Study Consortium for Acadia, Adamas, Addex, Aeonian, Alexza, Anavex, Arvelle Therapeutics, Inc, Axcella Health, Axovant, Biogen, Biomotiv/Koutif, Blackfynn, Bloom Science, Bridge Valley Ventures, Cavion, Cerebral Therapeutics, Cerevel, Clinilabs, Concert Pharmaceuticals, Covance, Crossject, CuroNZ, Eisai, Empatica, Encoded, Engage Therapeutics, Encoded, Epitel, GW Pharma, Idorsia, Impax, Ionis, J&J Pharmaceuticals, Marinus, MonosolRx, Neurelis, Neurocrine, Novartis, Otsuka Pharmaceutical Development, Ovid Therapeutics Inc, Pfizer, Pfizer‐Neusentis, Praxis, Redpin, Sage, Sancillio, Shire, SK Life Sciences, Springworks, Stoke, Sunovion, Supernus, Takeda, UCB Inc, Ultragenyx, Upsher‐Smith, Vyera, West Therapeutic Development, Xenon, Xeris, Zogenix, and Zynerba. Dr French has also received research grants from Biogen, Cavion, Eisai, Engage, GW Pharma, Lundbeck, Neurelis, Ovid, SK Life Sciences, Sunovion, UCB, and Zogenix as well as grants from the Epilepsy Research Foundation, Epilepsy Study Consortium, and NINDS (#U01‐NS038455). She is on the editorial board of Lancet Neurology and Neurology Today. She is scientific officer for the Epilepsy Foundation for which NYU receives salary support. She has received travel reimbursement related to research, advisory meetings, or presentation of results at scientific meetings from the Epilepsy Study Consortium, the Epilepsy Foundation, Adamas, Arvelle Therapeutics, Inc, Axovant, Biogen, Blackfynn, Crossject, CuroNz, Eisai, Engage, Idorsia, Neurelis, Novartis, Otsuka, Ovid, Pfizer, Redpin, Sage, SK Life Science, Sunovion, Takeda, UCB, Ultragenyx, and Zynerba. Dr Dlugos receives salary support from NIH, Commonwealth of Pennsylvania Department of Health, Pediatric Epilepsy Research Foundation, and The Epilepsy Study Consortium. He is an investigator on research grants awarded to CHOP from Zogenix, Greenwich Biosciences, Brain Sentinel, Neurelis, Q‐State, Aquestive, Bio‐Pharm, SK Life Sciences, and Encoded Therapeutics. He has received travel expenses for protocol development conferences or investigator meetings from Marinus, Ovid/Takeda, Ultragenyx, Pfizer, Biogen, BioMarin, and Xenon. He received honoraria and/or travel support for CME and other educational programs from Wake Forest University School of Medicine, American Epilepsy Society, American Academy of Neurology, Epilepsy Foundation of America, Epilepsy Foundation of North Carolina, Medscape, Miller Medical Communications, Ecuador Neurology Project, Ministry of Health of the United Arab Emirates, Seoul National University, and the Chinese Pediatric Society.

## ETHICAL PUBLICATION STATEMENT

We confirm that we have read the Journal's position on issues involved in ethical publication and affirm that this report is consistent with those guidelines.
